# Familial colorectal adenocarcinoma and hereditary nonpolyposis colorectal cancer: a nationwide epidemiological study from Sweden

**DOI:** 10.1054/bjoc.2000.1718

**Published:** 2001-04

**Authors:** K Hemminki, X Li

**Affiliations:** Department of Biosciences at Novum, Karolinska Institute, 141 57 Huddinge, Sweden

**Keywords:** familial risk, colorectal cancer, adenocarcinoma, endometrial cancer, right colon

## Abstract

Although estimates are available of the proportion of hereditary nonpolyposis colorectal cancer (HNPCC) among all colorectal cancer (CRC), its proportion among familial CRC is unclear. We estimated these proportions epidemiologically from the nationwide Swedish Family-Cancer Database on 9.6 million individuals. Colorectal adenocarcinomas were retrieved from the Cancer Registry covering years 1958–1996. Standardized incidence ratios (SIRs) were calculated for offspring (aged less than 62 years) when their parent had colorectal adenocarcinoma. In 9.82% of all families, an offspring and a parent were affected, giving a population attributable proportion of 4.91% and a familial SIR of 2.00. When offspring and parents shared the anatomic site, the SIR was 2.32 for proximal and 2.00 for distal CRC. When offspring were diagnosed before age 40 years and parents before age 50 years, the SIR was 25.72 for familial proximal CRC. In older age groups familial risks did not differ between proximal and distal CRC. Familial risks were increased also for endometrial, small intestinal and gastric cancers, manifestations in HNPCC. Depending on which assumptions were made, HNPCC was calculated to account for 20 to 50% of familial CRC, corresponding to 1 or 2.5% of all CRC among 0–61-year-old individuals. © 2001 Cancer Research Campaign http://www.bjcancer.com


*British Journal of Cancer* (2001) **84**(7), 969–974
© 2001 Cancer Research Campaign
doi: 10.1054/ bjoc.2001.1718, available online at http://www.idealibrary.com on IDEAL®

http://www.bjcancer.com

### Familial colorectal adenocarcinoma and hereditary nonpolyposis colorectal cancer: a nationwide epidemiological study from Sweden

**K Hemminki and X Li**

Department of Biosciences at Novum, Karolinska Institute, 141 57 Huddinge, Sweden

**Summary** Although estimates are available of the proportion of hereditary nonpolyposis colorectal cancer (HNPCC) among all colorectal cancer (CRC), its proportion among familial CRC is unclear. We estimated these proportions epidemiologically from the nationwide Swedish Family-Cancer Database on 9.6 million individuals. Colorectal adenocarcinomas were retrieved from the Cancer Registry covering years 1958–1996. Standardized incidence ratios (SIRs) were calculated for offspring (aged less than 62 years) when their parent had colorectal adenocarcinoma. In 9.82% of all families, an offspring and a parent were affected, giving a population attributable proportion of 4.91% and a familial SIR of 2.00. When offspring and parents shared the anatomic site, the SIR was 2.32 for proximal and 2.00 for distal CRC. When offspring were diagnosed before age 40 years and parents before age 50 years, the SIR was 25.72 for familial proximal CRC. In older age groups familial risks did not differ between proximal and distal CRC. Familial risks were increased also for endometrial, small intestinal and gastric cancers, manifestations in HNPCC. Depending on which assumptions were made, HNPCC was calculated to account for 20 to 50% of familial CRC, corresponding to 1 or 2.5% of all CRC among 0–61-year-old individuals. © 2001 Cancer Research Campaign http://www.bjcancer.com

**Keywords**: familial risk; colorectal cancer; adenocarcinoma; endometrial cancer; right colon

Colorectal cancer (CRC) is one of the commonest cancers in Western countries, with 5000 annual cases (over 10% of all cancers) in Sweden (IARC, 1990; Centre for Epidemiology, 1999). Over 90% of CRCs are adenocarcinomas but malignant carcinoids are relatively common at young age (Hemminki and Li, 2001). Most CRCs are sporadic and migrant studies have shown that environmental factors are important (Potter, 1999). At least 10% of the CRC burden has been suggested to be genetic (Lynch and de la Chapelle, 1999) and familial risks in population-based studies have varied between 1.7 and 2.0 (Fuchs et al, 1994; Carstensen et al, 1996; Hemminki et al, 1998; Hemminki and Vaittinen, 1999). According to a recent twin study, 60% of the variation in CRC was assigned to random environmental effects and 35% to heritable factors; shared environmental effects accounted for 5% which was not significant (Lichtenstein et al, 2000). The most common hereditary CRC disorder is hereditary nonpolyposis CRC (HNPCC), due to defective DNA mismatch repair (Wheeler et al, 2000). The estimates of the proportion HNPCC among all CRC have varied widely, depending on the criteria used and the population studied (Potter, 1999; Wheeler et al, 2000). In Finland, mutation-positive cases account for 3% of all CRC (Aaltonen et al, 1998; Salovaara et al, 2000). Because the relative risk of CRC is high in mutation carriers (cumulative incidence of CRC up to age 70 is 82%) compared to the general population (cumulative incidence 1.6), HNPCC may alone be responsible for a familial risk of 1.5 in Finland, i.e.: $0.82 \times 0.03/0.016 = 1.54$ (Aarnio et al, 1999). HNPCC is characterized by a dominant inheritance, relatively early onset (before age 50 years), proximal location of tumours (70% proximal to splenic flexture, compared to 30% in sporadic tumours), high penetrance and presentation of multiple CRCs and other cancers, particularly at the endometrium, small intestine, urinary tract, stomach and biliary system (Lynch and Smyrk, 1996; Aarnio et al, 1999; Lynch and de la Chapelle, 1999; Wheeler et al, 2000). Another hereditary cancer syndrome, familial adenomatous polyposis (FAP), is due to the mutations in the APC gene and accounts for less than 1% of all CRC (Vogelstein and Kinzler, 1998). Due to prophylactic colectomies and removal of precancerous lesions, the proportion of HNPCC and FAP is presumed to be decreasing among all CRC.

Familial clustering of CRC is thought to occur even when the cases are not part of a defined cancer syndrome (Fuchs et al, 1994; Hemminki et al, 2001; Dong and Hemminki, 2001). To investigate this, we analysed the risk of colorectal adenocarcinoma by family history using the nationwide Swedish Family-Cancer Database (Hemminki et al, 1998, 2001; Hemminki and Vaittinen, 1999). The database offers unique possibilities for reliable estimation of familial risks, because the data on family relationships and cancers were obtained from registered sources of practically complete coverage. We used the anatomic location of CRC, diagnostic age and other cancers in the family as clues to estimate the proportion of HNPCC among all familial cancers. We define familial cancer strictly as parental cancer so as to be compatible with the dominant mode of HNPCC inheritance. This study is the largest follow-up study of familial CRC and the only one solely on adenocarcinoma.

*Received 13 December 2000*
*Revised 23 January 2001*
*Accepted 24 January 2001*

*Correspondence to:* K Hemminki

### SUBJECTS AND METHODS

The Swedish Family-Cancer Database includes all persons born in Sweden after 1934 with their biological parents, totalling over 9.6

million individuals (Hemminki et al, 1999). Cancers were retrieved from the nationwide Swedish Cancer Registry from years 1958 to 1996. The completeness of colon and rectal cancer registration in the 1970s has been estimated to be 96.4% and 98.0%, respectively, and is now considered to be close to 100%. The percentage of cytologically or histologically verified cases by site, sex and age at diagnosis for colon and rectal cancers has been 98–99% (Centre for Epidemiology, 1999). The Family-Cancer Database has a gap among those born between 1935 and 1940 who died between 1960 and 1996. Many of these individuals lack links to parents in the Database and this probably causes a deficit of some cancers and a somewhat inflated risk estimates for fatal cancers. A 4-digit diagnostic code according to the 7th revision of the International Classification of Diseases (ICD-7) was used. The following ICD-7 codes were pooled: 'oral' cancer comprised codes 161 (larynx) and 140–148 (lip, mouth, pharynx), except for code 142 (salivary glands); 'lymphoma', comprised codes 200 (non-Hodgkin's lymphoma), 201 (Hodgkin's disease) and 202 (reticulosis); and 'leukaemia' comprised codes 204–207 (leukaemias), 208 (polycystaemia vera) and 209 (myelofibrosis). ICD codes 153.0 to 154.9, excluding 1541 for anus, were used for CRC. Based on the ICD codes, the anatomical location of CRC was classified as 'proximal' including right-sided sections, and 'distal' including left-sided sections of the colon and rectum; the splenic flexure was the dividing line of the proximal and distal locations. The histological classification of colorectal cancer was used to define adenocarcinoma, the only type of CRC considered in this study.

Family history information was collected on all first-degree relatives (parents, siblings, and children) but only the parent–offspring relationship was used in the present study, to cover only a dominant mode of inheritance. Moreover, families were counted only once even if more than one offspring or parent was affected. In case of more than one affected sibling, the first affected was selected. If both parents were affected, the father was selected. The Amsterdam criteria were considered among our classification of HNPCC (Vasen et al, 1991). Follow-up was started at birth or January 1958, whichever came latest. Follow-up was terminated on death, emigration, or the closing data of the study, December 31, 1996. Standardized incidence ratios (SIRs) were calculated as the ratio of observed (O) to expected (E) number of cases. The expected numbers were calculated from 5-year age-, period-, sex-specific incidence rates (Esteve et al, 1994). Confidence intervals (95%CI) were calculated assuming a Poisson distribution (Esteve et al, 1994).

The population attributable proportion of cases with a family history of CRC was estimated as follows: (familial SIR-1)/familial SIR × proportion of familial cases (Rothman and Greenland, 1998).

### RESULTS

The Family-Cancer Database included 3722 offspring and 63 698 parents with colorectal adenocarcinoma (Table 1). Proximal sites accounted for 29% and distal sites 61% of both parental and offspring cases, leaving some 10% of cases where location was 'other', including unspecified parts. The ratio of proximal to distal cases was 33:67 for offspring and parents. Familial cases accounted for 9.82% of offspring CRC.

The number of familial pairs is shown in Table 2 by anatomic site. Familial risks in offspring CRC were calculated for proximal and distal cancers. The number of proximal offspring–parent pairs was 36; mixed pairs numbered 124; distal pairs numbered 137. The distribution fits the binomial $(34\% + 66\%)^2$, suggesting that the offspring location of the tumour is independent of the parental location. When both offspring and parents presented with proximal CRC, the SIR was 2.32. Any other combination of sites resulted in SIRs of 1.87–2.00. The population attributable proportion of family history can be estimated from the familial SIR of 2.00 for all CRC, and the familial proportion of 9.82% giving 4.91%.

Familial risks in offspring were calculated by age at diagnosis (Table 3). Because of different age distributions in the offspring and parents, the age groups were divided at 40 and 50 years, respectively. In the youngest age group of proximal cancers, the SIR was 25.72, compared to the oldest group of proximal cancers

**Table 1** Number of colorectal adenocarcinoma cases by anatomic site and family history

| Colorectal adenocarcinoma subsites | Parents | Offspring | | |
|---|---|---|---|---|
| | All cases | All cases | Familial cases | Familial % |
| Proximal | 18604 (29.21%, 32.14%[a]) | 1076 (29.12%, 32.36%[a]) | 106 | 9.79 |
| Distal | 39281 (61.67%, 67.86%[a]) | 2249 (60.87%, 67.64%[a]) | 224 | 9.96 |
| Others | 5813 (9.12%) | 370 (10.01%) | 33 | 8.92 |
| All colorectum | 63698 (100.00%) | 3695 (100.00%) | 363 | 9.82 |

[a]% of the sum of proximal + distal cancers.

**Table 2** SIR for colorectal adenocarcinoma subsites in offspring by parental colorectal adenocarcinoma

| Parents's colorectal adenocarcinoma subsites | Offspring's colorectal adenocarcinoma subsites | | | | | | | | | | | | | | |
|---|---|---|---|---|---|---|---|---|---|---|---|---|---|---|---|
| | Proximal | | | | | Distal | | | | | All colorectum | | | | |
| | O | E | SIR | 95%CI | | O | E | SIR | 95%CI | | O | E | SIR | 95%CI | |
| Proximal | 36 | 15.55 | 2.32 | 1.62 | 3.13 | 63 | 33.73 | 1.87 | 1.43 | 2.36 | 110 | 54.90 | 2.00 | 1.65 | 2.40 |
| Distal | 61 | 31.66 | 1.93 | 1.47 | 2.44 | 137 | 68.43 | 2.00 | 1.68 | 2.35 | 217 | 111.51 | 1.95 | 1.70 | 2.21 |
| All colorectum | 106 | 51.52 | 2.06 | 1.68 | 2.47 | 224 | 111.46 | 2.01 | 1.76 | 2.28 | 363 | 181.58 | 2.00 | 1.80 | 2.21 |

 *© 2001 Cancer Research Campaign*

**Table 3** SIR for colorectal adenocarcinoma subsites in offspring by parental colorectal adenocarcinoma age at diagnosis

| Parent's colorectal adenocarcinoma subsites | Offspring's adenocarcinoma subsites: Proximal | | | | | | Distal | | | | | | All colorectum | | | | | |
|---|---|---|---|---|---|---|---|---|---|---|---|---|---|---|---|---|---|---|
| | <40 | | | ≥40 | | | <40 | | | ≥40 | | | <40 | | | ≥40 | | |
| | O | SIR | 95% CI | | O | SIR | 95% CI | | O | SIR | 95% CI | | O | SIR | 95% CI | | O | SIR | 95% CI |
| Proximal | | | | | | | | | | | | | | | | | | |
| <50 | 5 | **25.72** | **8.12 53.21** | 2 | 8.93 | 0.84 25.59 | 4 | **14.94** | **3.89 33.18** | 0 | | | 9 | **17.19** | **7.79 30.25** | 2 | **2.43** | **0.23 6.96** |
| ≥50 | 4 | 1.08 | 0.28 2.40 | 25 | **2.19** | **1.41 3.13** | 7 | 1.34 | 0.53 2.52 | 52 | **1.88** | **1.40 2.42** | 12 | 1.20 | 0.61 1.97 | 87 | **2.00** | **1.60 2.44** |
| All | 9 | **2.31** | **1.05 4.07** | 27 | **2.32** | **1.52 3.27** | 11 | 2.00 | 0.99 3.36 | 52 | **1.84** | **1.37** 2.38 | 21 | **1.99** | **1.23 2.93** | 89 | **2.01** | **1.61 2.45** |
| Distal | | | | | | | | | | | | | | | | | | |
| <50 | 7 | **16.33** | **6.47 30.68** | 2 | 3.75 | 0.35 10.74 | 4 | **6.77** | **1.76 15.04** | 3 | 2.44 | **0.46** 5.99 | 12 | **10.40** | **5.35 17.12** | 6 | **3.06** | **1.10 5.99** |
| ≥50 | 14 | 1.82 | 0.99 2.91 | 38 | **1.65** | **1.17 2.22** | 20 | **1.85** | **1.13 2.74** | 110 | **1.97** | **1.62 2.36** | 37 | **1.78** | **1.25 2.40** | 162 | **1.85** | **1.58 2.15** |
| All | 21 | **2.59** | **1.60 3.82** | 40 | **1.70** | **1.21 2.27** | 24 | **2.10** | **1.34 3.03** | 113 | **1.98** | **1.63 2.36** | 49 | **2.23** | **1.65 2.90** | 168 | **1.88** | **1.60 2.17** |
| All colorectum | | | | | | | | | | | | | | | | | | |
| <50 | 13 | **18.78** | **9.96 30.38** | 5 | **5.90** | **1.86 12.20** | 11 | **11.54** | **5.73 19.36** | 5 | 2.57 | **0.81** 5.31 | 25 | **13.41** | **8.67 19.19** | 12 | **3.85** | **1.98 6.34** |
| ≥50 | 20 | 1.61 | 0.98 2.39 | 68 | **1.81** | **1.41 2.27** | 30 | **1.71** | **1.15 2.38** | 178 | **1.96** | **1.68 2.25** | 54 | **1.60** | **1.20 2.06** | 272 | **1.90** | **1.68 2.14** |
| All | 33 | **2.52** | **1.73 3.45** | 73 | **1.90** | **1.49 2.36** | 41 | **2.22** | **1.59 2.95** | 183 | **1.97** | **1.69 2.26** | 79 | **2.22** | **1.76 2.74** | 284 | **1.95** | **1.73 2.18** |

Bold type: 95% CI does not include 1.00.

of 2.19. In the youngest age groups of proximal-distal and distal-proximal cancers the SIRs were 16.33 and 14.94, respectively. In the youngest age group of distal-distal cancers the SIR was 6.77. The oldest age groups in each pair of subsites showed uniform SIRs: proximal-proximal (2.19), proximal-distal (1.65), distal-proximal (1.88) and distal-distal (1.97). The diagnostic age of the offspring influenced the risks only when parents were diagnosed before age 50 years.

Offspring CRC risks were assessed by parental cancers in Table 4. Offspring were analysed separately when diagnosed <40 years of age. Offspring proximal CRC was increased when parents had gastric (borderline significance), small intestinal, endometrial, prostate and bone cancer, in addition to parental CRC. Most of these SIRs were higher when offspring were diagnosed before age 40; however, increases for small intestinal and prostate cancers were not significant and by bone cancer no cases were recoded. For distal CRC, with almost twice the number of cases compared to proximal sites, only parental CRC and endometrial cancers caused a significant increase. Early onset offspring CRC was increased by parental nervous system cancer.

Only 12 families of a total of 3.0 million families in the Database (0.0004%) fulfilled the Amsterdam criteria of 3 affected individuals. All 3 comprised 1 parent and 2 offspring. Of these 36, 18 (51%) were proximal and 17 (49%) were distal; the location of one could not be identified.

### DISCUSSION

The present overall familial risk of 2.00 for colorectal adenocarcinoma broadly agrees with the estimates from other population-based studies, taking into account our age limit of 61 years and the fact that the previous studies did not focus on a specific histology (Fuchs et al, 1994; Carstensen et al, 1996). The population attributable proportion for family history of 4.91% in the present study was also consistent with the 7% estimate from a US study on first-degree relatives because the inclusion of all first-degree relatives gives an exaggerated attributable proportion (Fuchs et al, 1994).

We characterized familial CRC in different ways, some of which are novel. The SIR for proximal CRC (2.32) was higher than that for distal CRC (2.00), particularly in younger individuals, (offspring <40 and parents <50) age groups (25.72 vs. 6.77). On the other hand, in the older age groups, anatomic site had no effect and SIRs remained below 2.0. The ratio among familial cases of proximal to distal sites was 34:66. The sum of the mixed sites (offspring proximal-parental distal + offspring distal-parental proximal) was exactly as predicted by a binomial distribution $(0.34 + 0.66)^2$. The anatomic location of familial CRC in offspring appeared thus to be largely independent of the CRC location in parents. The extracolonic sites which associated with CRC were endometrium, stomach, small intestine, prostate, bone and nervous system. However, only endometrial cancer associated with both proximal and distal CRC. The association with prostate cancer was of borderline significance.

HNPCC is the most common of the dominant hereditary CRC syndromes and its proportion among all CRC is an important question for cancer prevention and the search for novel susceptibility genes (Wheeler et al, 2000). The definition of HNPCC is based on the occurrence of CRC in family members, or more recently, on the demonstration of mutations in the mismatch repair genes. The widely used Amsterdam criteria require that CRC occurs in at least

© 2001 Cancer Research Campaign

3 close relatives in 2 or more generations (Boland, 1998). These criteria cannot be applied to our database because only 2 generations are available for studies on adult cancers. Thus only 12 HNPCC families were identified among a total of 3 million families. In other studies, applying the Amsterdam criteria, the proportion of HNPCC among all CRC has ranged from below 1 to 15% (Evans et al, 1997; Potter, 1999; Peel et al, 2000). In Finland, with 2 common founder mutations, the proportion of mutation-positive HNPCC cases is 3% (Salovaara et al, 2000). Such proportions depend on the study population, its age-structure and the frequency of founder mutations. More recently, mutation screening and prophylactic measures among families and diagnosed gene carriers may affect the population estimates. These factors appear to have caused a decrease in the frequency of FAP in the Swedish population. The diagnosis, defined as multiple synchronous CRCs, has disappeared from the Cancer Registry and, unless in situ cancers are considered, it is not possible to confirm any aggregation due to FAP. The Amsterdam criteria require that FAP be excluded. We assume that very little of our reported familial aggregation was due to FAP.

In contrast to FAP, several findings in the present study confirmed that we could identify HNPCC. High risks were observed at proximal sites and in the younger age groups, and at the extracolonic sites, including endometrium, stomach and small intestine cancers. HNPCC is assumed to affect proximal and distal colorectum in the ratio of 70:30, opposite to localization of sporadic tumours, 30:70 (Lynch and Smyrk, 1996; Lynch and de la Chapelle, 1999). If this were true, we could obtain a fairly accurate estimate on the proportion of HNPCC among all familial cases. Then the odds of finding an HNPCC tumour in a proximal compared to a distal site should be 5.4 (i.e, 70/30: 30/70). Similarly, according to a binomial distribution: $(0.70 + 0.30)^2 =$ 49% proximal-proximal sites, 42% proximal-distal or distal-proximal sites, and 9% distal-distal sites. Thus, 50% of the effects of HNPCC should be found at proximal sites, shared by offspring and parents for which the SIR was 2.32; conversely only 10% of the effect should be at distal sites, shared by offspring and parents, for which the SIR was 2.00. Such a small difference in observed SIR can at most accommodate a 20% aetiological proportion for HNPCC among all familial CRC. However, recent limited evidence, including data from the families fulfilling the Amsterdam criteria and mutation analysis, suggests that the 70:30 proximal-distal distribution of HNPCC may be too high and a 50:50 distribution may be more likely (Aaltonen et al, 1998; Peel et al, 2000; Salovaara et al, 2000).

Endometrial cancer in the CRC families provides an independent means of estimating the proportion of familial CRC due to HNPCC, based on the assumption that the increase in endometrial cancer in CRC families were only due to HNPCC and that the risk of endometrial cancer could be somewhat higher in HNPCC than that of CRC (Aarnio et al, 1999; Hemminki et al, 1999; Lynch and de la Chapelle, 1999; Wheeler et al, 2000). The SIR for offspring proximal CRC was equally high as the SIR for endometrial cancer, SIR 2.32; for distal CRC the increase (SIR 2.00, excess in SIR 1.00) was 2 times higher than that at endometrium (1.54, excess in SIR 0.54). Thus most proximal and close to 50% of distal CRC could be due to HNPCC. Because distal CRC is more than twice as common as proximal CRC, the above reasoning would suggest that some 50% of familial CRC were due to HNPCC. However, the association of gastric and small intestinal cancer with CRC was limited entirely to the proximal colon.

The above analyses were based on relatively small numbers, leaving substantial margins of error. Furthermore the data were based on indirect standardization (the SIR). Formally, SIRs from 2 populations are comparable only if the expected values were derived from the same population. As this was not strictly the case, small inaccuracies were probably introduced. The alternative approach of using direct standardization is tenuous with sparse data (Hemminki and Vaittinen, 1999).

These data are consistent with the notion that HNPCC is an important, probably predominant cause of CRC in the small group of young patients with proximal neoplasms but it has hardly any effect in the large group of older distal patients. Our 0–61-year-old population showed an overall familial risk of colorectal adenocarcinoma of 2.00 to offspring of parents with the same tumour. In 9.82% of families with an affected offspring, a parent was also affected, giving a population attributable proportion of 4.91. This can be compared to the Finnish estimate of mutation-positive HNPCC cases of 3% among all CRC. The incidence of CRC is lower in Finland than in Sweden, while Finnish HNPCC families are characterized by 2 common founder mutations that are rare in Sweden (Aaltonen et al, 1998; Salovaara et al, 2000), probably invalidating a direct comparison. Our best estimates for familial CRC due to HNPCC ranged from 20 to 50%. The overall proportion of HNPCC in CRC would range from 1% (20% of 5%) to 2.5% in the Swedish population aged 0–61 years. As most HNPCC cases occur before age 62, but only 1/5 of CRCs are diagnosed before that age (Centre for Epidemiology, 1999), the etiological proportion of HNPCC in the total population would be substantially lower than predicted above.

### ACKNOWLEDGEMENTS

Supported by the King Gustaf V Jubilee fund and The Cancer Fund.

### REFERENCES

Aaltonen L, Salovaara R, Kristo P, Canzzian F, Hemminki A, Peltomäki P, Chadwick R, Kääriäinen H, Eskelinen M, Järvinen H, Mecklin J-P and Chapelle De La A (1998) Incidence of hereditary nonpolyposis colorectal cancer and the feasibility of molecular screening for the disease. *N Engl J Med* **338**: 14811–14817

Aarnio M, Sankila R, Pukkala E, Salovaara R, Aaltonen L, De La Chapelle A, Peltomäki P, Mecklin J-P and Järvinen H (1999) Cancer risk in mutation carriers of DNA-mismatch-repair genes. *Int J Cancer* **81**: 214–218

Boland C (1998) Hereditary nonpolyposis colorectal cancer. In: Vogelstein B and Kinzler K (eds) *The Genetic Basis of Human Cancer* pp. 333–346. McGraw-Hill: New York

Carstensen B, Soll-Johanning H, Villadsen E, Söndergaard J and Lynge E (1996) Familial aggregation of colorectal cancer in the general population. *Int J Cancer* **68**: 428–435

Centre for Epidemiology (1999) Cancer Incidence in Sweden 1997 pp. 1–114: Stockholm

Dong, C and Hemminki K (2001) Modification of cancer risks in offspring by sibling and parental cancers from 2,112,616 nuclear families. *Int J Cancer*, in press

Esteve J, Benhamou E and Raymond L (1994) *Statistical Methods in Cancer Research* Vol. 128. IARC Scientific Publication. IARC: Lyon

Evans D, Walsh S, Jeacock J, Robinson C, Hadfield L, Davies D and Kingston R (1997) Incidence of hereditary non-polyposis colorectal cancer in a population-based study of 1137 consecutive cases of colorectal cancer. *Br J Surg* **84**: 1281–1285

Fuchs C, Giovannucci E, Colditz G, Hunter D, Speizer F and Willett W (1994) A prospective study of family history and the risk of colorectal cancer. *N Engl J Med* **331**: 1669–1674

 *© 2001 Cancer Research Campaign*

**Table 4** SIR for offspring colorectal adenocarcinoma subsites by parental cancer

| Parent's cancer | Proximal | | | | | | | | Distal | | | | | | | |
|---|---|---|---|---|---|---|---|---|---|---|---|---|---|---|---|---|
| | All ages | | | | <40 | | | | All ages | | | | <40 | | | |
| | O | SIR | 95% CI | | O | SIR | 95% CI | | O | SIR | 95% CI | | O | SIR | 95% CI | |
| No cancer | 559 | 0.85 | 0.78 | 0.92 | 185 | 0.85 | 0.73 | 0.98 | 1263 | 0.93 | 0.88 | 0.98 | 273 | 0.91 | 0.81 | 1.02 |
| Upper aerodigestive tract | 13 | 1.19 | 0.63 | 1.93 | 3 | 1.02 | 0.19 | 2.51 | 15 | 0.64 | 0.36 | 1.01 | 1 | 0.24 | 0.00 | 0.95 |
| Salivary glands | 2 | 1.77 | 0.17 | 5.07 | | | | | 3 | 1.24 | 0.23 | 3.04 | 2 | 4.60 | 0.43 | 13.18 |
| Oesophagus | 6 | 1.58 | 0.57 | 3.10 | 1 | 1.04 | 0.00 | 4.09 | 7 | 0.85 | 0.34 | 1.60 | 1 | 0.74 | 0.00 | 2.90 |
| Stomach | 37 | 1.41 | 0.99 | 1.90 | 12 | **1.99** | **1.02** | **3.28** | 51 | 0.88 | 0.66 | 1.14 | 9 | 1.06 | 0.48 | 1.86 |
| Small intestine | 6 | **3.01** | **1.08** | **5.90** | 2 | 3.70 | 0.35 | 10.60 | 5 | 1.17 | 0.37 | 2.43 | 1 | 1.32 | 0.00 | 5.16 |
| Colorectum | 112 | **2.03** | **1.67** | **2.43** | 34 | **2.44** | **1.69** | **3.33** | 233 | **1.95** | **1.71** | **2.21** | 43 | **2.19** | **1.58** | **2.89** |
| Adenocarcinoma | 106 | **2.06** | **1.68** | **2.47** | 33 | **2.52** | **1.73** | **3.45** | 224 | **2.01** | **1.76** | **2.28** | 41 | **2.20** | **1.58** | **2.93** |
| Proximal | 36 | **2.32** | **1.62** | **3.13** | 9 | **2.31** | **1.05** | **4.07** | 63 | **1.87** | **1.44** | **2.36** | 11 | 2.00 | 0.99 | 3.36 |
| Distal | 61 | **1.93** | **1.47** | **2.44** | 21 | **2.59** | **1.60** | **3.82** | 137 | **2.00** | **1.68** | **2.35** | 24 | **2.10** | **1.34** | **3.03** |
| Liver | 16 | 1.02 | 0.58 | 1.58 | 7 | 1.84 | 0.73 | 3.45 | 31 | 0.91 | 0.62 | 1.26 | 6 | 1.11 | 0.40 | 2.18 |
| Pancreas | 17 | 1.09 | 0.63 | 1.66 | 3 | 0.78 | 0.15 | 1.91 | 32 | 0.94 | 0.64 | 1.29 | 4 | 0.74 | 0.19 | 1.63 |
| Lung | 33 | 1.01 | 0.69 | 1.38 | 9 | 1.00 | 0.45 | 1.76 | 64 | 0.92 | 0.71 | 1.16 | 10 | 0.79 | 0.38 | 1.35 |
| Breast | 54 | 1.08 | 0.81 | 1.39 | 9 | 0.62 | 0.28 | 1.10 | 116 | 1.10 | 0.91 | 1.31 | 23 | 1.14 | 0.72 | 1.65 |
| Cervix | 12 | 1.07 | 0.55 | 1.76 | 3 | 0.92 | 0.17 | 2.25 | 22 | 0.93 | 0.58 | 1.36 | 6 | 1.31 | 0.47 | 2.56 |
| Endometrium | 32 | **2.32** | **1.59** | **3.20** | 12 | **3.14** | **1.61** | **5.16** | 45 | **1.54** | **1.12** | **2.02** | 12 | **2.23** | **1.15** | **3.67** |
| Ovary | 17 | 1.42 | 0.82 | 2.17 | 6 | 1.81 | 0.65 | 3.55 | 28 | 1.09 | 0.73 | 1.54 | 7 | 1.50 | 0.60 | 2.82 |
| Other female genitals | 1 | 0.46 | 0.00 | 1.80 | | | | | 7 | 1.46 | 0.58 | 2.75 | | | | |
| Prostate | 79 | **1.26** | **1.00** | **1.55** | 20 | 1.27 | 0.77 | 1.88 | 123 | 0.91 | 0.75 | 1.07 | 20 | 0.90 | 0.55 | 1.33 |
| Kidney | 21 | 1.29 | 0.80 | 1.90 | 1 | 0.23 | 0.00 | 0.91 | 38 | 1.08 | 0.77 | 1.46 | 6 | 0.99 | 0.35 | 1.93 |
| Urinary bladder | 29 | 1.35 | 0.90 | 1.88 | 6 | 1.05 | 0.38 | 2.06 | 56 | 1.21 | 0.91 | 1.55 | 7 | 0.87 | 0.34 | 1.63 |
| Melanoma | 7 | 0.77 | 0.31 | 1.45 | 2 | 0.66 | 0.06 | 1.89 | 25 | 1.34 | 0.87 | 1.92 | 8 | 1.90 | 0.81 | 3.44 |
| Skin | 12 | 0.80 | 0.41 | 1.32 | 6 | 1.68 | 0.61 | 3.30 | 26 | 0.80 | 0.52 | 1.13 | 4 | 0.79 | 0.21 | 1.76 |
| Eye | | | | | | | | | 3 | 1.08 | 0.20 | 2.64 | 2 | 3.92 | 0.37 | 11.23 |
| Nervous system | 16 | 1.27 | 0.72 | 1.96 | 5 | 1.32 | 0.42 | 2.74 | 35 | 1.32 | 0.92 | 1.79 | 13 | **2.46** | **1.30** | **3.97** |
| Thyroid gland | 4 | 1.14 | 0.30 | 2.53 | | | | | 6 | 0.81 | 0.29 | 1.59 | 1 | 0.69 | 0.00 | 2.72 |
| Endocrine glands | 8 | 1.06 | 0.45 | 1.91 | 3 | 1.32 | 0.25 | 3.24 | 16 | 1.01 | 0.57 | 1.56 | | | | |
| Bone | 4 | **5.87** | **1.53** | **13.03** | | | | | 4 | 2.78 | 0.72 | 6.17 | 1 | 3.60 | 0.00 | 14.10 |
| Connective tissue | 4 | 1.38 | 0.36 | 3.06 | 2 | 2.50 | 0.24 | 7.17 | 10 | 1.62 | 0.77 | 2.77 | 2 | 1.78 | 0.17 | 5.11 |
| Lymphoma | 11 | 0.76 | 0.38 | 1.28 | 3 | 0.75 | 0.14 | 1.84 | 26 | 0.84 | 0.55 | 1.20 | 3 | 0.53 | 0.10 | 1.31 |
| Myeloma | 10 | 1.34 | 0.64 | 2.30 | 3 | 1.61 | 0.30 | 3.94 | 17 | 1.05 | 0.61 | 1.61 | 4 | 1.52 | 0.39 | 3.37 |
| Leukaemia | 10 | 0.77 | 0.37 | 1.32 | 3 | 0.90 | 0.17 | 2.21 | 29 | 1.04 | 0.69 | 1.45 | 7 | 1.49 | 0.59 | 2.81 |
| Any cancer | 576 | **1.30** | **1.20** | **1.41** | 156 | **1.33** | **1.13** | **1.54** | 1078 | **1.13** | **1.07** | **1.20** | 204 | **1.23** | **1.07** | **1.41** |

Bold type: 95% CI does not include 1.00.

*© 2001 Cancer Research Campaign*

Hemminki K and Li X (2001) Increased cancer risk in offspring of women with colorectal carcinoma. A Swedish register-based cohort study. *Cancer*, **91**: 1061–1063

Hemminki K and Vaittinen P (1999) Familial cancers in a nation-wide family-cancer database: age distribution and prevalence. *Eur J Cancer* **35**: 1109–1111

Hemminki K, Vaittinen P and Kyyrönen P (1998) Age-specific familial risks in common cancers of the offspring. *Int J Cancer* **78**: 172–175

Hemminki K, Vaittinen P and Dong C (1999) Endometrial cancer in the Family-Cancer Database. *Cancer Epidemiol Biomarkers Prev* **8**: 1005–1010

Hemminki K, Vaittinen P, Dong C and Easton D (2001) Sibling risks in cancer: clues to recessive or X-linked genes? *Br J Cancer* **84**: 388–391

IARC (1990) *Cancer: causes, occurence and control* Vol. 100. IARC Scientific Publications. IARC: Lyon

Lichtenstein P, Holm N, Verkasalo P, Illiado A, Kaprio J, Koskenvuo M, Pukkala E, Skytthe A and Hemminki K (2000) Environmental and heritable factors in the causation of cancer. *N Engl J Med* **343**: 78–85

Lynch H and de la Chapelle A (1999) Genetic susceptibility to non-polyposis colorectal cancer. *J Med Genet* **36**: 801–818

Lynch H and Smyrk T (1996) Hereditary nonpolyposis colorectal cancer (Lynch syndrome). *Cancer* **78**: 1149–1167

Peel D, Ziogas A, Fox E, Gildea M, Laham B, Clements E, Kolodner R and Anton-Culver H (2000) Characterization of hereditary nonpolyposis colorectal cancer families from a population-based series of cases. *J Natl Cancer 1st* **92**: 1517–1522

Potter J (1999) Colorectal cancer: molecules and populations. *J Natl Cancer Inst* **91**: 916–932

Rothman K and Greenland S (1998) *Modern Epidemiology* Lippincott-Raven: Philadelphia

Salovaara R, Loukola A, Kristo P, Kaariainen H, Ahtola H, Eskelinen M, Harkonen N, Julkunen R, Kangas E, Ojala S, Tulikoura J, Valkamo E, Jarvinen H, Mecklin J, Aaltonen L and de la Chapelle A (2000) Population-based molecular detection of hereditary nonpolyposis colorectal cancer. *J Clin Oncol* **18**: 2193–2200

Vasen H, Mecklin J-P, Meera Khan P and Lynch H (1991) The international collaborative group on hereditary non-polyposis colorectal cancer (ICG-HNPCC). *Diseases Colon Rectum* **34**: 424–425

Vogelstein B and Kinzler KW (1998) The genetic basis of human cancer. McGraw-Hill: New York

Wheeler J, Bodmer W and Mortensen N (2000) DNA mismatch repair genes and colorectal cancer. *Gut* **47**: 148–153

*© 2001 Cancer Research Campaign*
